# A combined machine for collecting and chopping rice straw

**DOI:** 10.1016/j.heliyon.2022.e10412

**Published:** 2022-08-28

**Authors:** Mahmoud Awad, Osama Fouda, Wael Fathy, Wael El Balkemy, Mohsen Egela, Walied El-Fakhrany, Mahmoud Okasha

**Affiliations:** Agricultural Engineering Research Institute (AEnRI), Agricultural Research Center (ARC), Giza 12611, Egypt

**Keywords:** Rice straw, Chopper, Collecting efficiency, Chopping quality

## Abstract

Due to the recent high prices of livestock feed in the world and the new sustainable management practices of rice straw, livestock farmers are obligated to either use or purchase rice straw and cut it to desired lengths to feed ruminants. Therefore, this study aimed to construct a combined machine for collecting and chopping rice straw for lengths preferred in feeding ruminants. The combined machine comprises three main units: a picking up unit, a chopping unit, and a takeout unit. Field experiments were performed on average rice straw moisture content of 25% (w.b.) to evaluate the performance of the combined machine, under factors of 1.3, 1.6, 1.9, and 2.2 km h^−1^ forward speeds, elevator velocities of 0.79, 0.94, and 1.10 m s^−1^, and chopping knives rotational speeds of 1600, 2000 and 2400 rpm. The obtained results indicated that the consumed specific energy was 90.94 kWh ha^−1^ to achieve the highest field capacity of 0.24 ha h^−1^, with collecting efficiency of 95.30% at a forward speed of 2.2 km h^−1^, elevator velocity of 1.10 m s^−1^ and rotational speed of 2400 rpm for chopping knives. In order to achieve the highest chopping quality of 95%, it is recommended to operate the elevator velocity at 0.79 m s^−1^, rotate chopping knives at 2400 rpm, and drive the tractor at a forward speed of 1.3 km h^−1^. The total operating cost of the combined machine is lower than the traditional methods by about 49.84%.

## 1 Introduction

Egypt is considered the highest rice producer in Africa, with a planting area of about 0.55 million hectares with a predicted yield of approximately 4.89 million metric tons (FAOSTAT 2020). Rice straw is a byproduct of rice harvesting left in the field behind combine harvesters. It can be gathered, burned, or left to degrade naturally. Rice straw is a residue that causes major environmental problems, as burning it emits carbon dioxide and ash, while buried waste can cause issues associated with eutrophication (Torregrosa et al., 2021). Experimentally, IRRI (2019) cleared that the ratio of straw/grain ranged from 0.5 to 0.7 throughout the experiment. Rice straw is lignocellulosic biomass composed of 38% cellulose, 25% hemicellulose, and 12% lignin (Japan Institute of Energy - Asian biomass handbook, 2002).

Nonetheless, it is regarded as a possible feed supplement for boosting protein and energy content. The consumption limit of rice straw for ruminants is 1.25 kg per 100 kg live mass per day (Drake et al., 2002). Straw's high silica and lignin concentration contribute to its low digestion of nutrients (dry matter and protein) (<50%). Thus, pretreatment of straw with urea at a 2–4% concentration is essential to maximize its contribution to improving milk and meat production (Gummert et al., 2020; Aquino et al., 2016).

In Bangladesh, Nipa et al. (2021) constructed and improved a fodder chopping machine tested on straw, grass, and maize. The chopping efficiency ranged from 93 to 96%, machine productivity ranged from 192 to 600 kg h^−1^, and energy consumption ranged from 0.0025 to 0.01 kWh for the different fodder. The evaluation results of straw combine “chopper and spreader units” revealed that chopped straw size and fuel consumption were 80–120 mm and 5.5–6.0 l h^−1^, respectively, with an average field capacity of 0.45 ha h^−1^ at 2.5–3.0 km h^−1^ forward speed and 1600–2000 rpm cylinder speed (Bhavya et al., 2020). On the other side, Ramulu et al. (2020) used the above technique, which recorded that the residue management machine's fuel consumption, field capacity, and field efficiency were 12.5–14.0 l h^−1^, 0.43–0.64 ha h^−1^, and 60.46%, respectively. Zheng et al. (2017) conformed studies on an integrated machine to pick up, crush and bury the corn straw in a single operation. As sstated in the field experiment, the straw picking up rate was 93.5%, the qualification rate of straw chopping length was 92.6%. Khairy et al. (2015) developed a machine for chopping straw depending on rice straw's physical properties. Abdrabo and Abdalh (2013) modified a John Deere-based pickup baler for chopping and compressing rice straw and stalks. The first found that the straw picking up rate and the qualification rate of chopping lengths were 93.5% and 92.6%, respectively, at 3.0 km h^−1^ operating speed. But, the second concluded maximum productivity and optimum specific energy requirement of 6.03 kg h^−1^ and 52.08 kWh at 1600 rpm drum speed. The third revealed that increasing the forward speed decreases cut lengths of rice straw (63–32 mm) and increases effective field capacity, decreasing energy consumption and operating costs.

Ismail (2012a) used response surface methods (RSM) that provided statistical tools for designing and analyzing experiments to optimize the performance of pickup machines. The RSM spotlights a bright spot during the final stages of process development, where the high pickup machine of in-specification parameters can be achieved with minimal operation. At the same time, Ismail et al. (2012b,c) studied the influence of the same variables on straw chopped factor percentage, average chopped straw length, and power requirements. The results showed that the highest straw cutting factor percentage was 99.60%, achieved at 4.46 m s^−1^ chopped unit rotation speed. The cutter drums interference was adjusted at 10 mm when the cutting disc was 25 mm. The length of the cutting straw and the power requirements recorded 24.90 mm and 44.03 kW of these parameters, respectively. Ismail et al. (2009) recorded the best value of straw elevated quantity was 7.075 kg min^−1^, attained at a combined units rotation speed of 102 rpm, and straw feed rate of 4 kg min^−1^. Increasing the straw holder's heights from zero to 2 cm increases the field capacity from 0.058 to 0.086 fed h^−1^, decreasing the chassis tilt angles from 36 to 28°.

Regarding rice straw cutting length, Ghaly et al. (2013) stated that the length of straw ranged from 14.49 to 18.85 mm, relying on the cutting speed and cutting depth. On the other side, El-Sayed et al. (2013) and El-Khateeb (2001) reported that increasing cutter speed from 500 to 1500 rpm increases the percentage of cutting length in the range (0.2 cm) by (54–93%). Meanwhile, El-Khateeb and El-Keway (2012) found that the maximum percentage of ‏>‏ 2 cm cutting length of 94% was recorded at 1500 rpm cutting speed for 22% (w.b.) rice straw moisture content. Hegazy et al. (2021) modified and tested a Star forage chopper machine (SFCM) to reduce the power required and improve forage cutting efficiency. The performance of SFCM was evaluated based on its ability to chop rice straw at different feed rates and knife speeds. The minimum cut length was 13.5 mm and was achieved by using a feed rate of 1.5 t h^−1^ with 136.2 m s^−1^ knife speed using toothed blades. Lotfy (2003) found an average cut length was 2.8 cm for cutting rice straw. But, El-Iraqi and El-Khawaja (2003) mentioned that the maximum percentage in cutting length of less than 5 cm recorded 87.8%, at 10.09 m s^−1^ cutting speed, 0.77 ton h^−1^ feeding rate, and 1.5 mm knife clearance. Kamel et al. (2003) found that the cost was 22.07 LE fed^−1^ (12.46 LE ton^−1^) when chopping rice straw at 0.53 m s^−1^ forward speed and 13.49% straw moisture content with 12 cutter head knives. The clearance between two knives and cutting drum speed affects maximum productivity (2.85 kg min^−1^ at 4 mm knives clearance and 18.70 m s^−1^ cutting drum speed), as indicated by Arif and Elaiwa (2009), who modified a machine for cutting rice straw.

According to the previous studies, a suitable machine remains needed and required to chop rice straw left in the field behind the combine harvesters into desirable uniform lengths of about 15–30 mm and sizing for feeding livestock. It is essential to perform two operations (smoothly picking up and chopping rice straw) with quick action and efficient cutting. Therefore, this research aims to construct a combined machine to collect, chop, and reserve the cutting rice straw in a large tank in one pass and determine the optimum parameters that produce a desirable straw length for livestock feed.

## 2 Materials and methods

### 2.1 The combined machine

**Figure 1:**
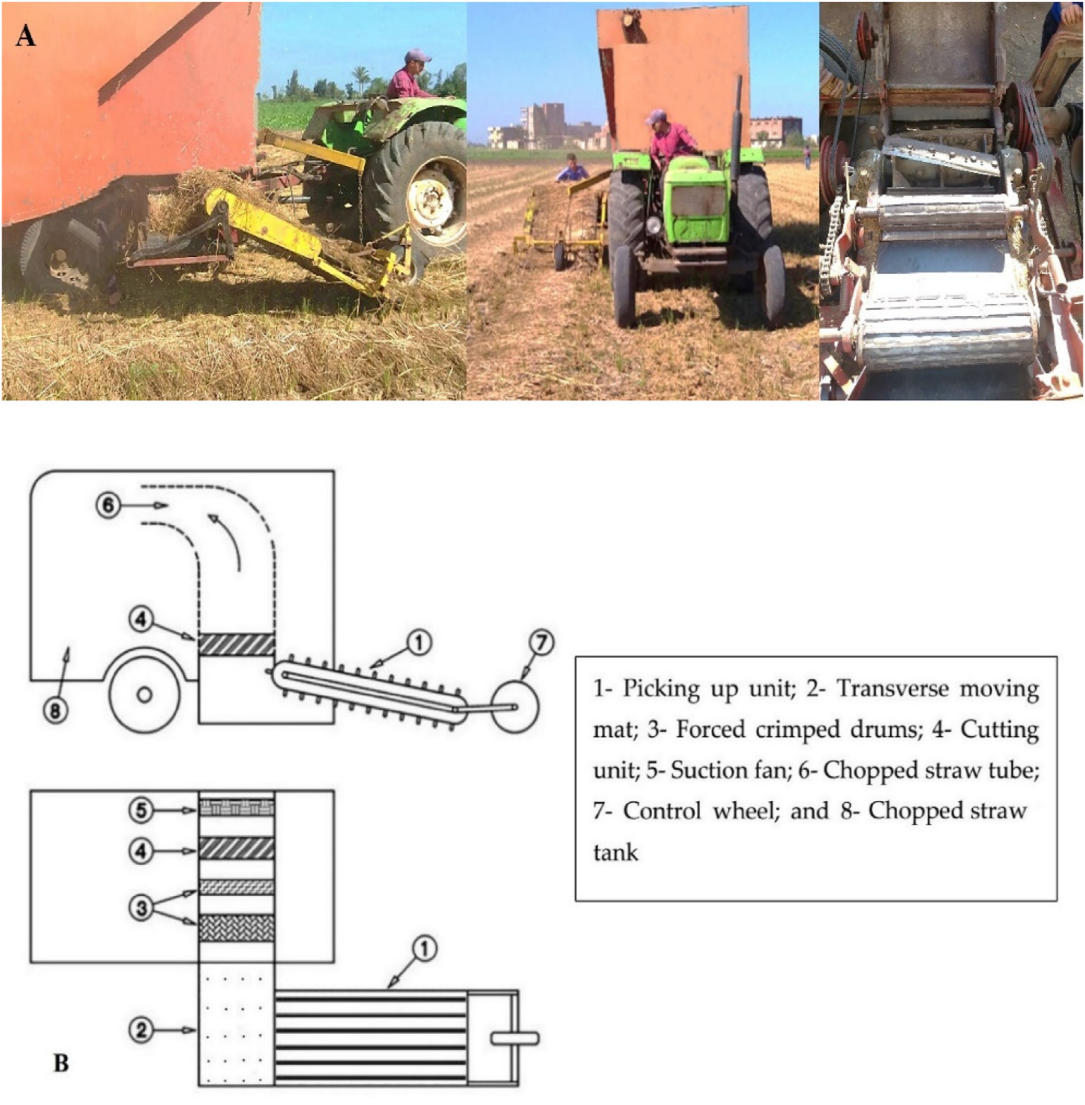
(A) Photos and (B) diagram of the combined machine.

The combined machine was manufactured in a private (Abd-Elsatar Kabeel) workshop at Kafr-Snjab village, El-Dakahlia Governorate, Egypt. The combined machine consists of three main units. The machines’ units are arranged as follows; picking up unit, chopping unit, and chopped straw tank, as shown in Figure 1. The combined machine was developed to be pulled by a tractor with adjustable picking up the height by a control wheel. Collecting rice straw in the field using the picking up unit, when the picker is operating, the fingers that are fixed on six conveyor belts move in the inclined direction to complete the picking, pushing, and backward throwing of the straw towards the transverse moving mat and the crimped drums. The transverse moving mat and the crimped drums divert the loose straw towards the chopping unit as a straw guiding element. The chopping unit consisted of a cylindrical chamber with a fixed knife and a drum supply with high-speed rotating knives to grip the stalks and make a cut at desired lengths. The chopped straw undergoes compression as it passes to the output unit by a high-speed suction fan to suck up the chopped straw and push it into the chopped straw tank.

#### 2.1.1 Picking up unit

**Figure 2:**
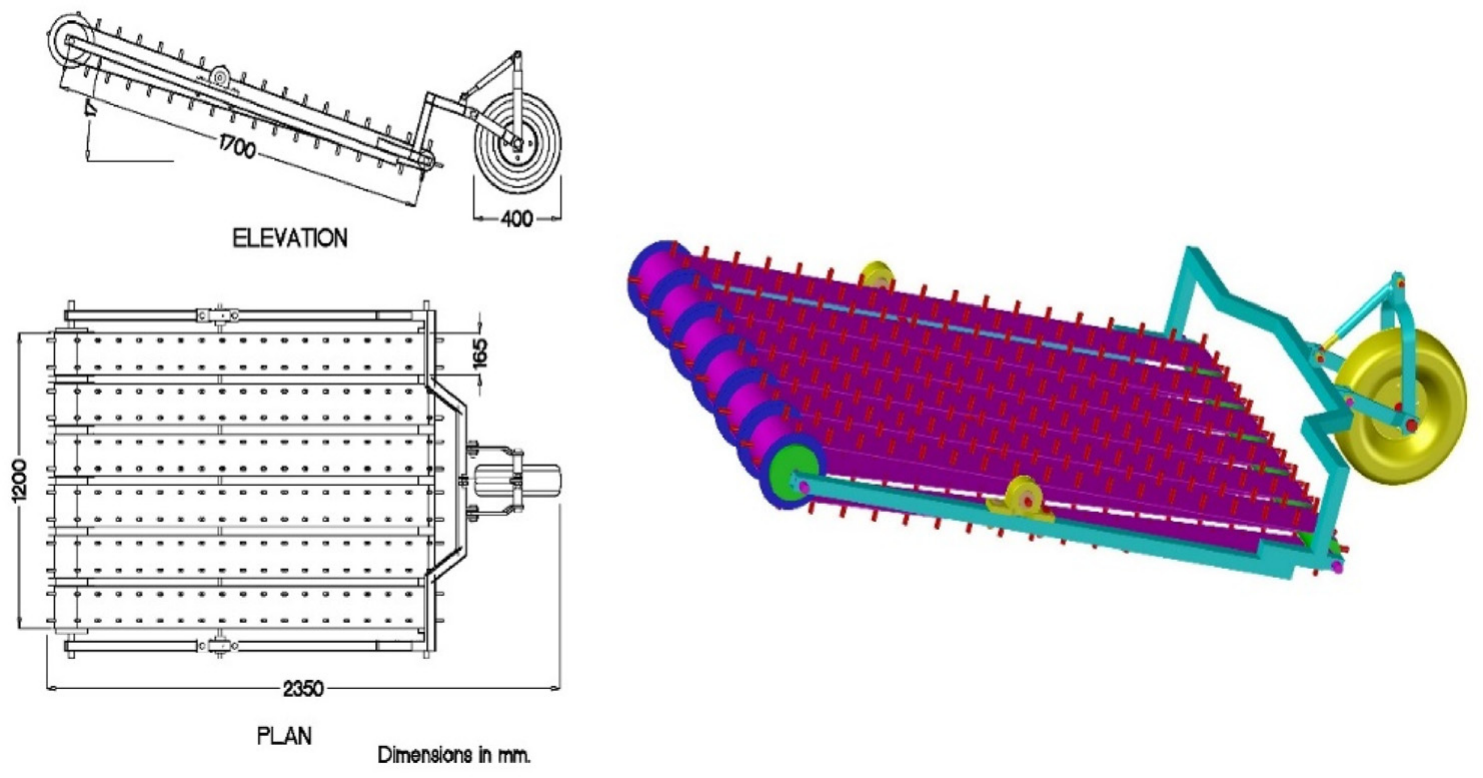
The picking up unit.

The picking up unit consists of a control wheel located in the front, then the picking device, which includes 216 fingers with 4.0 and 1.0 cm for length and diameter, respectively, and they were fixed on six conveyor belts each of 16.5 cm width. The collecting device has an inclined angle of 17° with the soil surface. A transverse moving mat with 65 cm width and two crimped drums receives rice straw from the picking device and directs it to the chopping unit, as shown in Figures 1 and 2.

#### 2.1.2 Chopping unit

**Figure 3:**
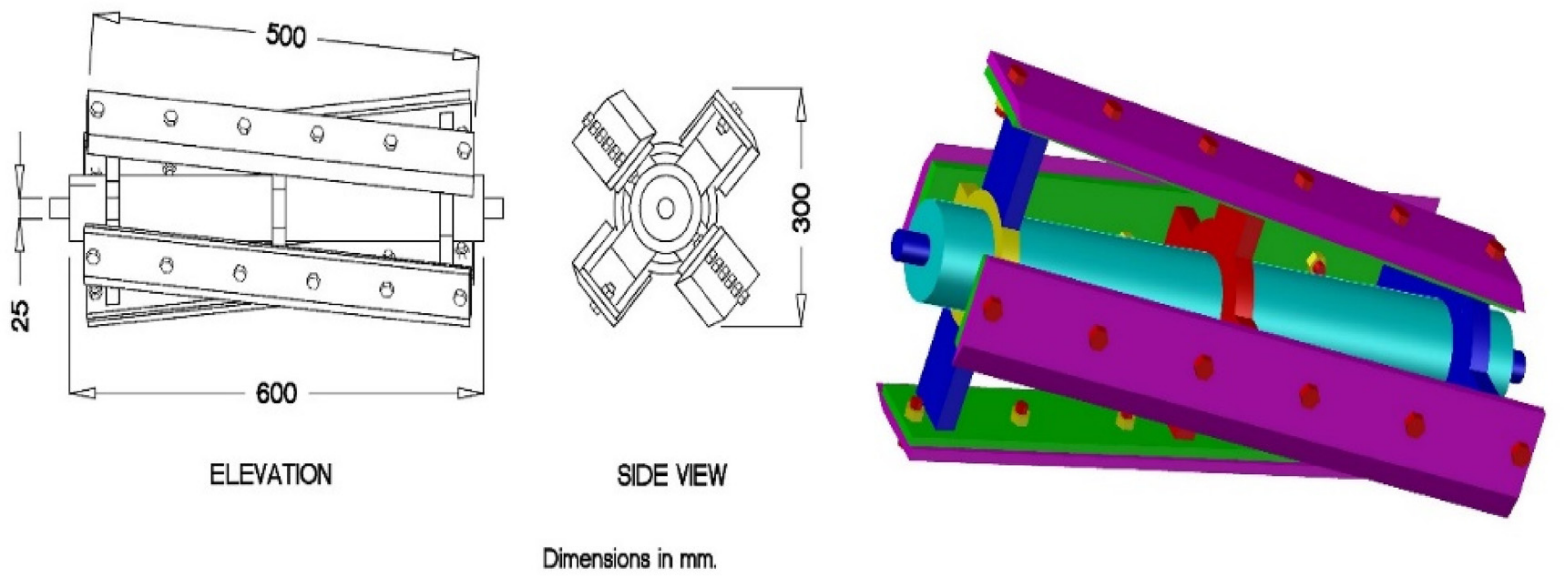
The cutting knives drum.

The cutting device includes a cylindrical chamber with a fixed knife and a drum supply with high-speed rotating knives, as shown in Figure 3. Both fixed and rotating knives have the exact dimensions of 50 and 10 cm for length and width, respectively. The cutting angle between the fixed and rotating knife was about 8°. There is no clearance between the fixed knife and the fourth rotating knives. Also, the rotating knives drum with its high-speed assists in pushing chopped rice straw to the output unit. The specific dimensions are shown in Figure 3.

#### 2.1.3 Takeout unit

**Figure 4:**
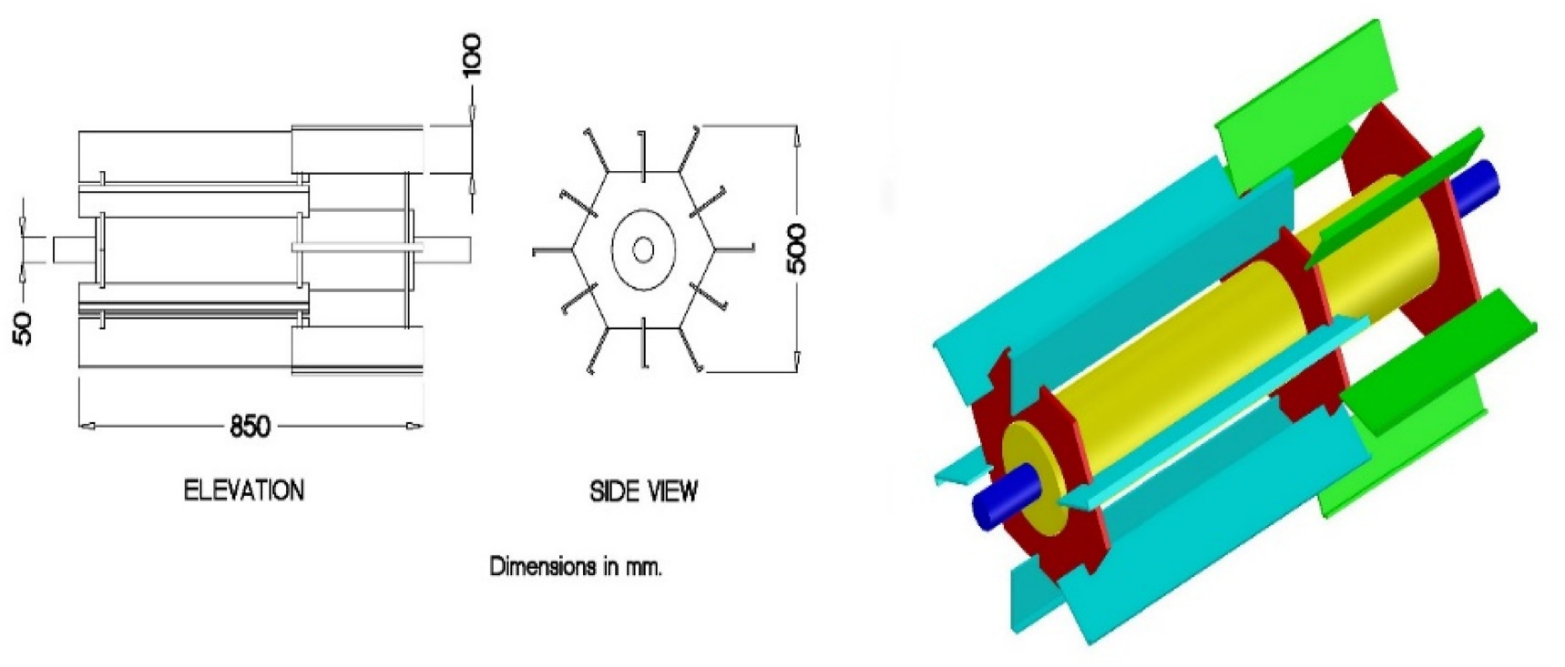
The takeout high-speed fan.

The takeout unit has a high-speed fan of 2400 rpm, as shown in Figure 4, making a high suction to suck up the chopped straw and push it into the chopped straw tank. The chopped straw tank had a dimension of 4.0 m length, 2.5 m width, and 3.0 m height, with a capacity of 1.0 ton for storing the chopped rice straw during the operation in the field.

### 2.2 Power source

The combined machine is mounted and powered by a 45-kW tractor (Deutz-Fahr, 2WD) with a fuel tank capacity of 56 L and a liquid-cooled system. The tractor's power take-off speed was 540 rpm during the operation of the combined machine in all treatments.

### 2.3 Rice straw properties

The properties of rice straw during chopping time were determined. Rice straw samples were randomly selected to measure the moisture content based on the American Society of Agricultural Engineers (ASAE) standard 358-1-2 with the support of laboratory tools to conduct the required measurements of rice straw materials. Average of moisture content (MC) of 25%)w.b. ((SD = ±1.34 and CV = 5.36%) was recorded, average stem length of rice straw was 99.6 cm (SD = ±2.98 and CV = 3%) and average of stem diameter at base, center, and top were 5.47, 3.99, and 1.87 mm, SD = ± (0.48, 0.28, and 0.08) and CV = (8.8, 7.1, and 4.4%), respectively.

### 2.4 Field experiments

The field trials were executed at Kafr-Snjab village, El-Dakahlia Governorate (31°22′60″ E, longitude; 31°02′60″ N, latitude; and 11.21 m height) in northeast Egypt. The combined machine was evaluated against its collecting efficiency (CE), chopping quality (CQ), actual field capacity (FC), specific energy (SE), and operating cost. Tests were arranged at four forward speeds (FS) of 1.3, 1.6, 1.9, and 2.2 km h^−1^, three elevator velocities (ES) of 0.79, 0.94, and 1.10 m s^−1^, three rotational speeds of 1600, 2000, and 2400 rpm for cutting knives drum (CKS), plus three replications for each treatment. The experimental area was divided into three main blocks to carry out 108 treatments in a split split-plot design. Each main block was divided into 36 plots; every plot had 100 m length and 1.2 m width. Twenty-five random samples of rice straw were selected to represent the sample size from each block of the experimental field. The data were statistically analyzed by regression analysis and mathematically achieved using Microsoft Excel program 2016 version 1807.

### 2.5 Measurements

The collection efficiency (CE) of rice straw was calculated according to Eq. (1), as follows:

(1) $$CE=\;\frac{St-Sr}{St}\;x\;100$$

where CE is collection efficiency (%), St is the total mass of rice straw (kg) (St = Sc + Sr), Sr is the total mass of uncollected rice straw after finishing operation (kg), and Sc is the total mass of the collected rice straw in the straw tank (kg).

Egyptian farmers use rice straw for feeding livestock after cutting it into approximate lengths of about 15–30 mm to achieve the animals’ preference chaff sizes, according to Khader (1997). Therefore, the chopping quality (CQ) was calculated according to Eq. (2), as follows:

(2) $$CQ=\;\frac{M1}{M}\;x\;100\;$$

where CQ is chopping quality, M1 is the mass of the lengths ≥15 mm and ≤30 mm (g), and M is the total mass of the sample (g). The representative sample for all different experimental treatments was 1000 g of the chopping rice straw.

The actual field capacity (FC) was determined according to Suliman et al. (2003), as follows in Eq. (3).

(3) $$FC=\frac{60}{At}\;\left(ha\;h^{-1}\right)$$

where FC is actual field capacity (ha h^−1^), At is total actual operation time per hectare (min ha^−1^) (At = Nt + Tt + Pt), Nt is maintenance and lubrication time (min ha^−1^), Tt is turning time (min ha^−1^), and Pt is parasitic time (min ha^−1^).

The specific energy (SE) was estimated by estimating the fuel consumption (fu) used to operate the combined machine. Fuel consumption was measured according to Elsbaay and Hegazy (2016), and then the following equation was used to calculate SE according to Hunt (1983), as follows in Eq. (4).

(4) $$SE=\left(\frac{fu\times \rho f\times LCV}{3600}\right)\times \left(\frac{427\times \eta th\times \eta mec}{75\times 1.36\times FC}\right)$$

where SE is specific energy (kWh ha^−1^), f_u_ is fuel consumption (l h^−1^), ρf is fuel density (0.85 kg l^−1^ for diesel), LCV is lower calorific value of fuel (1000 kcal kg^−1^), 427 is thermo-mechanical equivalent (J kcal^−1^), ηth is engine thermal efficiency (≈35% for diesel engines), ηmec is engine mechanical efficiency (≈80% for diesel engines), and FC is actual field capacity (ha h^−1^).

The hourly operating cost for operating the combined machine was estimated using the following equations according to Awady (1978), as follows in Eq. (5).

(5) $$C=\frac{p}{h}\left(\frac{1}{y}+\frac{i}{2}+t+r\right)+\left(a\times w\times s\times f\right)+\left(\frac{m}{144}\right)$$

where C is hourly operating cost ($ h^−1^), p is the price of the machine ($), h is yearly operating hours for the machine (h per year), y is the machine life expectancy (years), i is interest rate per year for the machine (%), t is taxes rate for the machine (%), r is repair and maintenance ratio for the machine (%), a is ratio of rated power and lubrication related to fuel cost (1.2), w is power consumption during operation (kW), s is specific fuel consumption (l kW^−1^h^−1^), f is fuel price ($ l^−1^), m is operator's monthly wage ($ per month), and 144 is monthly working hours. Operating cost per unit area was estimated by dividing the hourly operating cost by the actual field capacity as follows in Eq. (6).

(6) $$Operating\;cost\;\left(\$\;ha^{-1}\right)=\frac{hourly\;operating\;cost\;\left(\$\;h^{-1}\right)}{actual\;field\;capacity\;\left(ha\;h^{-1}\right)}$$

### 2.6 Statistical analyzes

The data were statistically analyzed by regression analysis and mathematically achieved using Microsoft Excel program 2016 version 1807. Analysis of variance with interactions was done using XLSTAT software (statistical add-in for Microsoft Excel) version 2022 (Addinsoft Inc., New York, USA).

## 3 Results and discussion

### 3.1 Field data

#### 3.1.1 Effect of forward speed, elevator velocity and rotational speed for chopping knives on collecting efficiency

**Figure 5:**
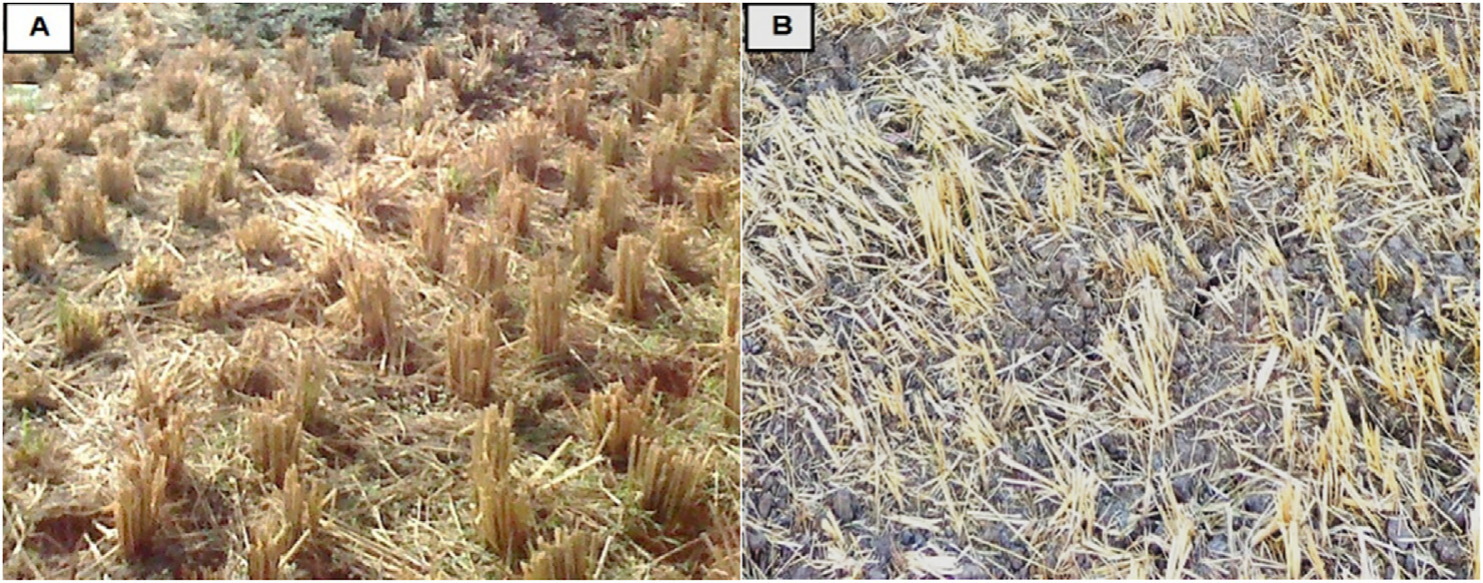
The uncut portion of rice straw after harvest (the stubble); manual transplanting (A), and direct seeding (B).

**Figure 6:**
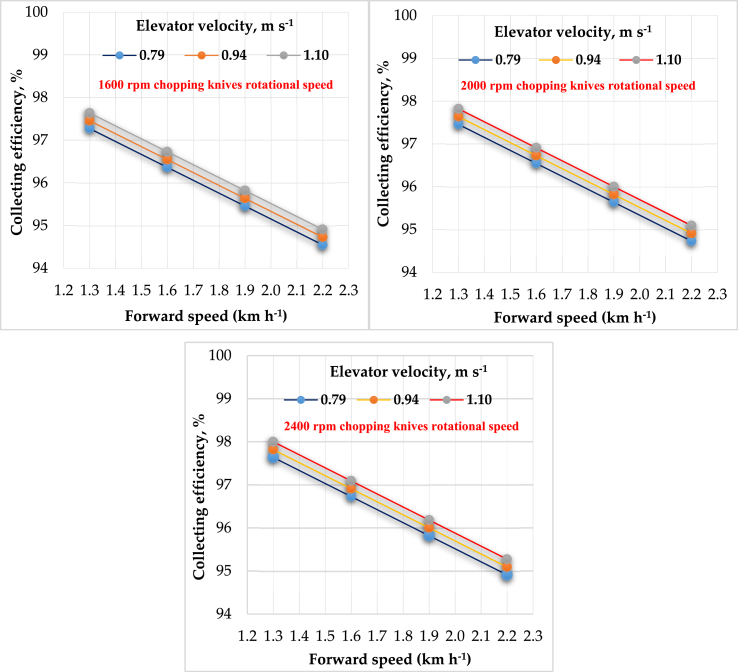
Effect of forward speeds on collecting efficiency at different elevator velocities for different cutting knives speeds.

In the beginning, it is necessary to state that the field conditions represented in the stubble (the uncut portion of rice straw after harvest), which ranged in height from 8 to 12 cm, acted as a carrier for the harvested rice straw and prevented it from sticking to the soil surface, as shown in Figure 5. Based on the above, this made the collector device operation easier and raised its efficiency. Data analyzed revealed that there was a significant effect of the forward speeds (FS) (p < 0.05), a highly significant effect for elevator velocities (ES) (p < 0.0001), and a highly significant effect for cutting knives speeds “CKS” (p < 0.01) on the collecting efficiency. Table 1 and Figure 6 show the effect of forward speeds on collection efficiency at different elevator velocities for different cutting knives speeds.

**Table 1:** Effect of forward speed, elevator velocity and knives drum speed on collecting efficiency, chopping quality, actual field capacity and specific energy.

	Response variable (measurements)											
	Collecting efficiency, %			Chopping quality, %			Actual field capacity, fed h^−1^			Specific energy, kWh ha^−1^		
Elevator velocity, m s^−1^	0.79	0.94	1.10	0.79	0.94	1.10	0.79	0.94	1.10	0.79	0.94	1.10
Forward speed, km h^−1^	Knives drum speed, 1600 rpm			Knives drum speed, 1600 rpm			Knives drum speed, 1600 rpm			Knives drum speed, 1600 rpm		
1.30	97.27	97.45	97.64	75.00	70.00	65.00	0.14	0.16	0.17	106.74	98.77	94.44
1.60	96.36	96.55	96.73	70.00	65.00	60.00	0.17	0.17	0.18	100.83	97.03	93.20
1.90	95.45	95.64	95.82	65.00	60.00	55.00	0.19	0.20	0.21	97.09	95.34	92.26
2.20	94.55	94.73	94.91	60.00	55.00	50.00	0.21	0.22	0.23	96.26	93.99	91.61
	Knives drum speed, 2000 rpm			Knives drum speed, 2000 rpm			Knives drum speed, 2000 rpm			Knives drum speed, 2000 rpm		
1.30	97.45	97.64	97.82	90.00	85.00	80.00	0.15	0.16	0.17	104.08	97.48	93.04
1.60	96.55	96.73	96.91	85.00	80.00	75.00	0.17	0.18	0.19	99.78	96.08	92.30
1.90	95.64	95.82	96.00	80.00	75.00	70.00	0.19	0.20	0.21	96.28	93.35	91.69
2.20	94.73	94.91	95.09	75.00	70.00	65.00	0.21	0.22	0.23	95.28	92.49	91.12
	Knives drum speed, 2400 rpm			Knives drum speed, 2400 rpm			Knives drum speed, 2400 rpm			Knives drum speed, 2400 rpm		
1.30	97.64	97.82	98.00	95.00	90.00	85.00	0.15	0.16	0.17	101.66	96.15	92.04
1.60	96.73	96.91	97.09	90.00	85.00	80.00	0.17	0.18	0.19	98.88	95.08	91.20
1.90	95.82	96.00	96.18	85.00	80.00	75.00	0.19	0.21	0.21	95.82	92.06	91.09
2.20	94.91	95.09	95.27	80.00	75.00	70.00	0.21	0.22	0.23	94.65	91.78	90.94

The relationship between collection efficiency and forward speeds has an inverse proportion, but it has a direct proportion with both elevator velocities and cutting knives speeds. The highest efficiency of collecting rice straw was 98% at a forward speed of 1.3 km h^−1^, elevator velocity of 1.10 m s^−1^, and cutting knives speed of 2400 rpm. The results are consistent with many studies as the straw picking up rate was 93.5% (Zheng et al., 2017), also Abdrabo and Abdalh (2013) stated that the first found that the straw picking up rate and the qualification rate of chopping lengths were 93.5%. Generally, the collection efficiency increased by increasing elevator velocities and cutting knives speeds and decreased by increasing the forward speeds. These results may be because of the increase in forward speed increasing the area of picking, which increases the amount of picked straw, subsequently increasing the burden on the picking device and decreasing its performance. The multiple regression analysis shows the effect of forward speeds (F), elevator velocities (E) and cutting knives speeds (C) on collecting efficiency (CE) by the following equation, which illustrates the relation as shown in Eq. (7):

(7) CE = 99.53 – 3.02F + 1.17 E + 0.00046 C R^2^ = 97%

The regression analysis declares that elevator velocities and cutting knives speeds are directly proportional to collecting efficiency but are inversely proportional to forward speeds. The factors that affected the (CE) are arranged in the following ascending order relative to the analysis of variance. Elevator velocity (the p-value from analysis as P_v1_ = 6.7 × 10^−5^) > cutting knives speed (the p-value from analysis as P_v2_ = 10^−4^) > forward speed (the p-value from analysis as P_v3_ = 2 × 10^−2^). Based on the Type III sum of squares, the following variables bring significant information to explain the variability of the dependent variable collecting efficiency: forward speeds, elevator velocities × cutting knives speeds, while the other variables do not bring significant information. The forward speed variable is the most influential among the explanatory variables based on the Type III sum of squares.

#### 3.1.2 Effect of forward speed, elevator velocity and rotational speed for chopping knives on chopping quality

**Figure 7:**
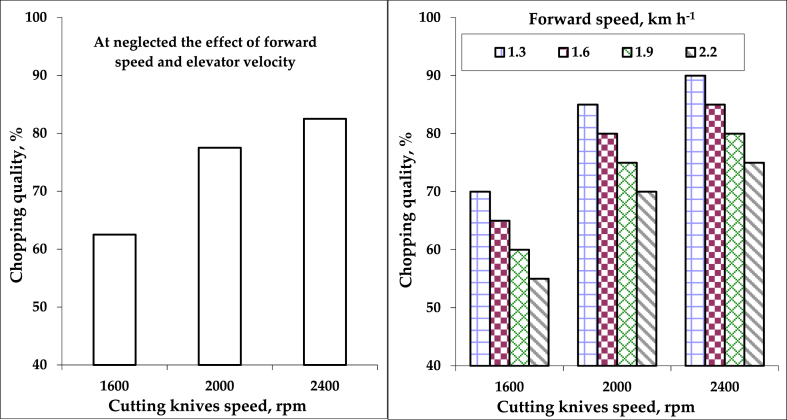
Effect of different cutting knives speeds on chopping quality neglected the effect of forward and elevator velocities.

**Figure 8:**
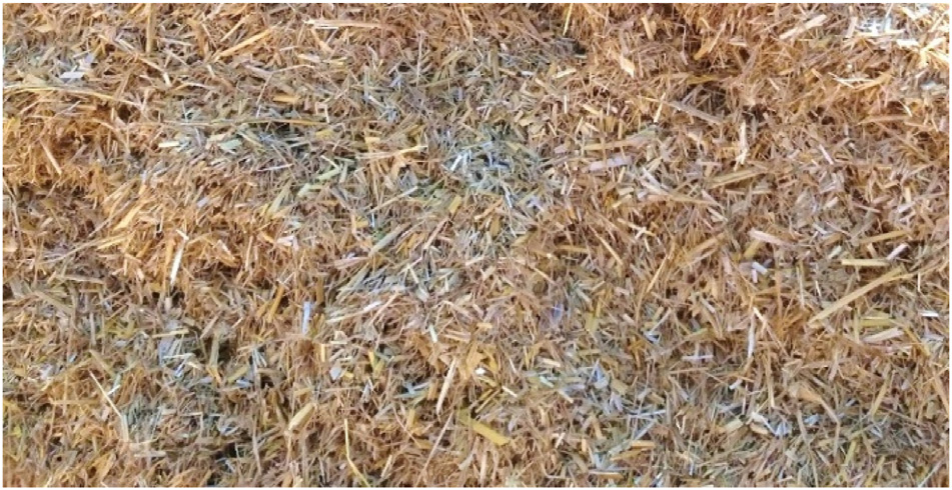
The animals' preference chaff sizes into a uniform length ≥15 mm and ≤30 mm.

Data analyzed showed a highly significant effect of the cutting knives speeds, but there is a non-significant effect on chopping quality for both forward and elevator velocities. This may be due to the large size of the machine, in which the straw tank can hold one ton and difficult field conditions, which led to a limited choice between slow speeds, which did not exceed 2.2 km h^−1^. Therefore, neglecting the effect of forward speeds and elevator velocities to focus on the effect of the cutting knives speeds on the chopping quality, as shown in Table 1 and Figure 7. Obviously, by increasing the cutting knives speeds, the chopping quality increased. It was increased by about 32% by increasing the cutting knives speed from 1600 to 2400 rpm. The obtained results were similar to data reported by Tian et al. (2021). This result may be attributed to increasing the rotating speed of cutting knives, increasing the number of cutting hits of rice straw per unit time, achieving the animals’ preference chaff sizes into a uniform length ≥15 mm and ≤30 mm, as shown in Figure 8. The multiple regression analysis shows the effect of forward speeds (F), elevator velocities (E), and cutting knives speeds (C) on chopping quality (CQ) by the following equation, which illustrates the relation, as shown in Eq. (8):

(8) CQ = 83.33 – 16.67 F – 31.85 E + 0.025 C R^2^ = 79%

The regression analysis reveals that elevator velocities and cutting knives speeds are directly proportional to chopping quality, but inversely proportional to forward speeds. The factors that affected the (CQ) are arranged in the following ascending order relative to the analysis of variance. Cutting knives speed (the p-value from analysis as P_v1_ = 9.24 × 10^−8^) > elevator velocity (the p-value from analysis as P_v2_ = 0.154) > forward speed (the p-value from analysis as P_v3_ = 0.607). Based on the Type III sum of squares, the following variables bring significant information to explain the variability of the dependent variable chopping quality: cutting knives speeds, forward speeds × elevator velocities, while the other variables do not bring significant information. Among the explanatory variables, the cutting knives speed variable is the most influential based on the Type III sum of squares.

#### 3.1.3 Effect of forward speed, elevator velocity and rotational speed for chopping knives on actual field capacity

**Figure 9:**
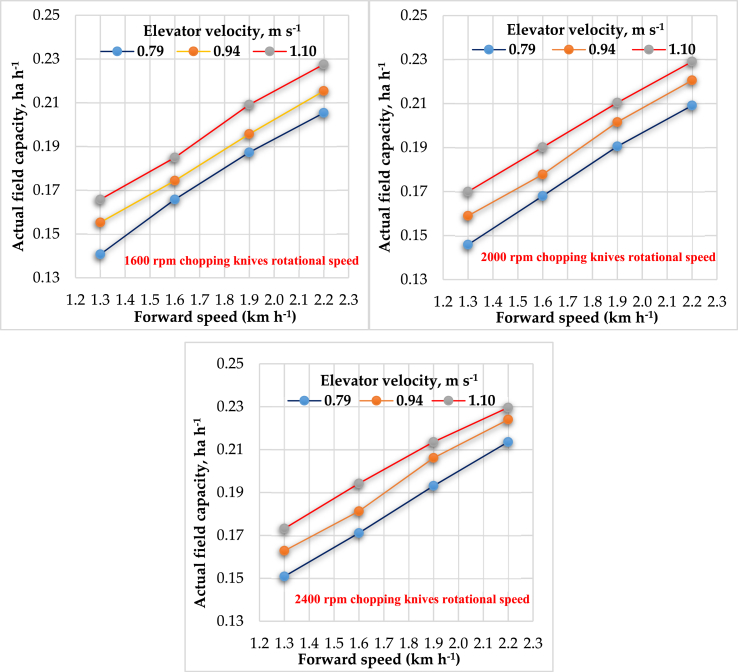
Effect of forward speeds on actual field capacity at different elevator velocities for different cutting knives speeds.

The relationship between the actual field capacity and machines’ forward speeds with different elevator velocities for different cutting knives speeds is shown in Table 1 and Figure 9. Forward speeds, elevator velocities, and cutting knives speeds directly affect actual field capacity; the actual field capacity increased by increasing forward speeds, elevator velocities, and cutting knives speeds. The results indicated that the highest value of actual field capacity was 0.23 ha h^−1^, obtained at 2.20 km h^−1^ forward speed, elevator velocity of 1.10 m s^−1^ at 2400 rpm cutting knives speed. These results were being consistent with Abdrabo and Abdalh (2013), Bhavya et al. (2020), and Ramulu et al. (2020). In contrast, the lowest value of actual field capacity was 0.15 ha h^−1^ at a forward speed of 1.30 km h^−1^, elevator velocity of 0.79 m s^−1^, and 1600 rpm cutting knives speed. The multiple regression analysis shows the effect of forward speeds (F), elevator velocity (E) and cutting knives speeds (C) on actual field capacity (FC) by the following equation, which illustrates the relation as shown in Eq. (9):

(9) FC = -0.0126 + 0.068 F + 0.068 E + 0.0000088 C R^2^ = 98.5%

The regression analysis shows that forward speeds, elevator velocity, and cutting knives speeds are directly proportional to actual field capacity. The factors that affected the (FC) are arranged in the following ascending order relative to the analysis of variance. Forward speed (the p-value from analysis as P_v1_ = 2.22 × 10^−38^) > elevator velocity (the p-value from analysis as P_v2_ = 3.42 × 10^−25^) > cutting knives speed (the p-value from analysis as P_v3_ = 5.74 × 10^−7^). Based on the Type III sum of squares, the following variables bring significant information to explain the variability of the dependent variable actual field capacity: forward speeds, elevator velocities, while the other variables do not bring significant information. Based on the Type III sum of squares, the forward speed variable is the most influential among the explanatory variables.

#### 3.1.4 Effect of forward speed, elevator velocity and rotational speed for chopping knives on specific energy

**Figure 10:**
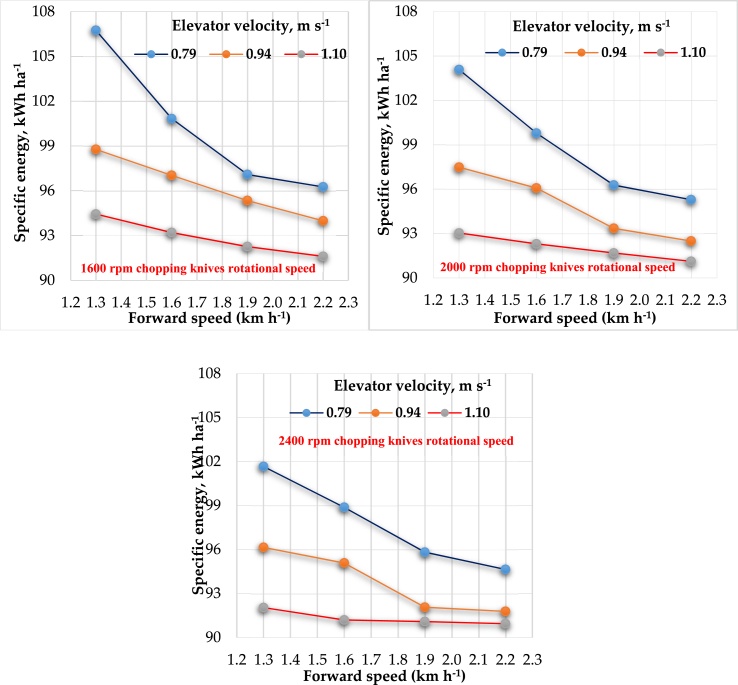
Effect of forward speeds on the specific energy at different elevator velocities for different cutting knives speeds.

Table 1 and Figure 10 show the relationship between the forward speeds and the specific energy at different elevator velocities for different cutting knives speeds. In general, by increasing the forward speed, elevator velocity, and cutting knives speed, the specific energy decreased. This result could be ascribed to an increase in the dynamic collecting area, by increasing the forward speed, elevator velocity and cutting knives speed led to an increase in the chopped rice straw; thus, large areas can be collected and chopped in a short time, led to decrease the power required per unit area in a specified period. The highest value of specific energy was 106.74 kWh ha^−1^ achieved at an operational elevator velocity of 0.79 m s^−1^ at a forward speed of 1.3 km h^−1^ and a cutting knives speed of 2400 rpm. Energy required was being consistent with Ismail et al. (2012b,c) and Nipa et al. (2021). However, the lowest value was 90.94 kWh ha^−1^ at an operational forward speed of 2.2 km h^−1^, elevator velocity of 1.10 m s^−1^, and cutting knives speed of 2400 rpm. The multiple regression analysis displays the effect of forward speeds (F), elevator velocity (E), and cutting knives speeds (C) on specific energy (SE) by the following equation, which illustrates the relation as shown in Eq. (10):

(10) SE = 131.65 – 5.86 F – 21.87 E – 0.00273 C R^2^ = 92.3%

The regression analysis declares that forward, elevator velocities, and cutting knives speeds are inversely proportional with (SE). The factors that affected the (SE) are arranged in the following ascending order relative to the analysis of variance. Elevator velocity (the p-value from analysis as P_v2_ = 1.37 × 10^−13^) > forward speed (the p-value from analysis as P_v1_ = 5.14 × 10^−11^) > cutting knives speed (the p-value from analysis as P_v3_ = 1.8 × 10^−3^). Finally, the data analyzed showed a significant effect for forward speed, elevator velocity, cutting knives speed, and (p < 0.001) on the specific energy. Based on the Type III sum of squares, the following variables bring significant information to explain the variability of the dependent variable specific energy: forward speeds × elevator velocities, elevator velocities × cutting knives speeds, while the other variables do not bring significant information. Based on the Type III sum of squares, forward speed × elevator velocities is the most influential among the explanatory variables.

### 3.2 Cost estimation

An economic estimation was performed for the combined machine and the traditional method and compared the costs for both systems.

#### 3.2.1 Traditional method

This method involves three main operations, which were assumed, as follows: (1) cost of picking up the straw from the field and compressing it into bales, ≈ $45 per ha, (2) cost of transporting the bales to the side of the field or the bunds, ≈ $30 per ha, and (3) cost of chopping the straw by the stationary chopper, ≈ $130 per ha. Consequently, the total operating cost for the traditional method is about $205 per ha.

#### 3.2.2 The combined machine

The total cost of the tractor and the combined machine includes two cost categories: fixed costs and variable costs, according to (Awady 1978).

Table 2 shows the most important assumptions as pre-mentioned in Eq. (5) for calculating the total operating cost for the tractor (as a power source), and the combined machine. Based on the assumptions, the total operating cost for the combined machine is $102.82 per ha, at 0.23 ha h^−1^ effective field capacity. Consequently, the total operating cost for the combined machine is lower than the cost of the traditional method by about 49.84%.

**Table 2:** Suggested cost criteria for the tractor and the combined machine.

Category	Symbol	Tractor	Combined machine
Price of machine ($)	p	15000	9000
Working hours (h per year)	h	1500	200
Life expecting of the machine (year)	y	20	10
Interest on the cost of the machine (%)	i	10%	10%
Taxes rate (%)S	t	17%	17%
Repair and maintenance (%) from the fixed cost	r	10%	5%
Rated power and lubrication ratio related to fuel cost	a	1.2	-
Power consumption (kW)	w	45	-
Specific fuel consumption (l kW^−1^h^−1^)	s	0.14	-
Fuel price, $ per l	f	0.44	-
Operator's monthly wage, $ per month	m	160	160

## 4 Conclusion

An integrated combined machine for collecting and chopping rice straw was developed and manufactured with functions to collect, chop, and reserve the cutting rice straw in a large tank in one pass. It has the characteristics of unrestricted, simple structure, reliable performance, easy operation, and high efficiency. The machine adopts a picking up unit with fixed fingers on six conveyor belts to complete the straw picking operation with desired collecting efficiency of 95.8%. In the collecting and chopping rice straw machine, a transverse moving mat and crimped drums diverted the stand loose straw towards the chopping unit as a straw guiding element. The chopping unit consisted of a cylindrical chamber with a fixed knife and a drum supply with high-speed rotating knives to grip the stalks and make straw uniform length ≥15 mm and ≤30 mm and a size corresponding to preferred straw sizes for animals with chopping quality of 85%. The chopped straw passes to the output unit by a high-speed suction fan to suck up the chopped straw and push it into the chopped straw tank. The developed machine can achieve a field capacity of 0.193 ha h^−1^ and it needs specific energy of 95.82 kWh ha^−1^ when it is operated with 1.9 km h^−1^ forward speed, 0.79 m s^−1^ for elevator velocity, and using high cutting knives speed of 2400 rpm for cutting the straw as a recommended combination set of the operating parameters.

## Declarations

### Author contribution statement

Mahmoud Awad; Wael El Balkemy: Conceived and designed the experiments; Contributed reagents, materials, analysis tools or data.

Osama Fouda: Conceived and designed the experiments; Performed the experiments; Contributed reagents, materials, analysis tools or data; Wrote the paper.

Wael Fathy: Performed the experiments; Analyzed and interpreted the data; Wrote the paper.

Mohsen Egela: Analyzed and interpreted the data; Contributed reagents, materials, analysis tools or data.

Walied El-Fakhrany: Conceived and designed the experiments; Contributed reagents, materials, analysis tools or data; Wrote the paper.

Mahmoud Okasha: Performed the experiments; Contributed reagents, materials, analysis tools or data; Analyzed and interpreted the data; Wrote the paper.

### Funding statement

This research did not receive any specific grant from funding agencies in the public, commercial, or not-for-profit sectors.

### Data availability statement

Data will be made available on request.

### Declaration of interests statement

The authors declare no conflict of interest.

### Additional information

No additional information is available for this paper.
